# Lactate normalization within 6 hours of bundle therapy and 24 hours of delayed achievement were associated with 28-day mortality in septic shock patients

**DOI:** 10.1371/journal.pone.0217857

**Published:** 2019-06-03

**Authors:** Seung Mok Ryoo, Ryeok Ahn, Tae Gun Shin, You Hwan Jo, Sung Phil Chung, Jin Ho Beom, Sung-Hyuk Choi, Young -Hoon Yoon, Byuk Sung Ko, Hui Jai Lee, Gil Joon Suh, Won Young Kim, for the Korean Shock Society (KoSS) Investigators

**Affiliations:** 1 Department of Emergency Medicine, University of Ulsan College of Medicine, Asan Medical Center, Seoul, Republic of Korea; 2 Department of Emergency Medicine, University of Ulsan College of Medicine, Ulsan University Hospital, Ulsan, Republic of Korea; 3 Department of Emergency Medicine, Samsung Medical Center, Sungkyunkwan University School of Medicine, Seoul, Republic of Korea; 4 Department of Emergency Medicine, Seoul National University Bundang Hospital, Seongnam, Republic of Korea; 5 Department of Emergency Medicine, Yonsei University College of Medicine, Seoul, Republic of Korea; 6 Department of Emergency Medicine, Guro Hospital, Korea University Medical Center, Seoul, Republic of Korea; 7 Department of Emergency Medicine, College of Medicine, Hanyang University, Seoul, Republic of Korea; 8 Department of Emergency Medicine, Seoul National University Boramae Medical Center, Seoul, Republic of Korea; 9 Department of Emergency Medicine, Seoul National University College of Medicine, Seoul, Republic of Korea; Azienda Ospedaliero Universitaria Careggi, ITALY

## Abstract

This study evaluated the prognostic ability of lactate normalization achieved within 6 and 24 h from septic shock recognition. Data from a septic shock registry from October 2015 to February 2017 were reviewed. The study included 2,102 eligible septic shock patients to analyze the prognostic ability of lactate normalization, defined as a follow-up lactate level <2 mmol/L within six hours of bundle therapy and within 24 hours of delayed normalization. The primary outcome was 28-day mortality. The overall 28-day mortality rate was 21.4%. The rates of lactate normalization within 6 and 24 h were significantly higher in the survivor groups than in the non-survivor group (42.4% vs. 23.4% and 60.2% vs. 31.2%; P<0.001, respectively). Multivariate logistic regression analysis showed that both 6- and 24-h lactate normalization were independent predictors (odds ratio [OR] 0.58, 95% confidence interval [CI] 0.45–0.75, p<0.001 and OR 0.42, 95% CI 0.33–0.54, p<0.001, respectively). When we could not achieve the lactate normalization, the sensitivity, specificity, positive, and negative predictive value to predict mortality were 76.6%, 42.4%, 26.5% and 87.0% respectively for 6-h normalization, and 68.8%, 60.2%, 32.0% and 87.7% respectively for 24-h normalization. Besides 6-h lactate normalization, 24-h delayed lactate normalization was associated with decreasing mortality in septic shock patients. Lactate normalization may have a role in early risk stratification and as a therapeutic target.

## Introduction

Hyperlactatemia in sepsis and septic shock occurs as a result of tissue hypoxia, accelerated aerobic glycolysis driven by excess beta-adrenergic stimulation, or other cause. Regardless of the source, increased lactate levels are associated with a poor prognosis.[1] Lactic acidosis can cause reduced cardiac contractility and vascular hypo-responsiveness to vasopressors through various mechanisms.[1] Moreover, in normotensive patients with sepsis, lactate concentrations above 4 mmol/L were independently correlated with a higher mortality rate and, therefore, require urgent recognition and proper resuscitation.[2] Additionally, high initial lactate level as well as prolonged time to lactate normalization was also associated with increased mortality.[3] Previous reports have demonstrated improved outcomes in patients with severe sepsis and septic shock who achieved both lactate clearance and lactate normalization.[4–6] Since 2013, Surviving Sepsis Campaign (SSC) guidelines have recommended bundle therapy for patients with sepsis and septic shock, including measurements of lactate level within 3 h and re-measurement at 6 h in cases with elevated initial lactate level.[7]

A significant reduction in mortality was seen in lactate-guided resuscitation compared to that in resuscitation without lactate monitoring (relative risk [RR] 0.67; 95% CI, 0.53–0.84; low quality) in five randomized controlled trials including a total of 647 patients.[5,8–11] The SSC recommended lactate normalization as a target of resuscitation in patients with elevated lactate level, with a goal to achieve normalization as rapidly as possible.[12]

Early risk stratification and prognostication in patients with septic shock are important because high-risk patients may benefit from earlier clinical interventions, whereas low-risk patients may benefit from avoiding unnecessary procedures. Although a previous study showed that both initial lactate level and normalization time were significantly associated with an increased risk of death in intensive care unit (ICU) patients,[3] the prognostic value of lactate normalization in septic shock patients has not been fully established, especially regarding delayed lactate normalization.

Therefore, the present study assessed the prognostic ability of lactate normalization achieved within 6 and 24 h from shock recognition in septic shock patients treated with protocol-driven resuscitation bundle therapy at emergency departments (EDs).

## Materials and methods

### Setting and study population

This multicenter prospective, observational, registry-based study used data from the Korean Shock Society (KoSS septic shock registry) registry. The KoSS, a multicenter clinical research consortium for septic shock, was organized in 2013 and its investigators have prospectively collected data from septic shock patients at the EDs of 10 teaching hospitals throughout South Korea from October 2015. The institutional review board of Asan Medical Center [2015–1253] and each institution (Korea University Anam and Guro Hospital [HRPC2016-184], [KUGH15358-001], Samsung Medical Center [SMC2015-09-057], Yonsei University College of Medicine Severance Hospital [4-2015-0929], Gangnam Severance Hospital [3-2015-0227], Seoul National University Bundang Hospital [B-1409/266-401], Seoul National University College of Medicine [J-1408-003-599], Seoul National University Boramae Medical Center [16-2014-36], Hanyang University Hospital [HYUH2015-11-013-007], and Hallym University College of Medicine Gangnam Sacred Heart Hospital [2015-11-142]) approved the study protocol, and written informed consent was obtained before data collection.

Adult (aged ≥18 years) septic shock patients, defined as suspected or confirmed infection and evidence of refractory hypotension or hypoperfusion, were enrolled in the registry.[13–15] Refractory hypotension was defined as persistent hypotension (systolic blood pressure [SBP] <90 mmHg, mean arterial pressure [MAP] <70 mmHg, or an SBP decrease of >40 mmHg after adequate intravenous fluid challenge [20–30 mL/kg or at least 1 L or more of crystalloid solution administered over 30 min]) or as the need for vasopressors after fluid resuscitation.[16] Hypoperfusion was defined as a serum lactate concentration of 4 mmol/L or greater.[7] Patients who signed a “Do Not Attempt Resuscitation” (DNAR) order, met the inclusion criteria 6 h after ED arrival, were transferred from other hospitals without meeting the inclusion criteria upon ED arrival, or were directly transferred from ED to other hospitals were not enrolled in the KoSS septic shock registry. The case report form; standard definitions of 200 variables including clinical characteristics, therapeutic interventions, and outcomes of patients with septic shock; and an investigator manual were developed based on a literature review and a consensus of the study investigators. Data were collected via a standardized registry form and was entered into a web-based electronic database registry. Outliers or incorrect values were primarily filtered by this data entry system. Each site principal investigator also had a designated local research coordinator responsible for ensuring the accuracy of the data entry and verifying records. The quality management committee (QMC) consisting of emergency physicians, local research coordinators, and investigators in each ED was organized to regularly monitor and review the data quality. The QMC provided feedback to the research coordinators and investigators based on the results of the quality management process through the query function in the system or directly by phone to clarify data.

### Data collection

KoSS septic shock registry data were collected from October 2015 to December 2017. Demographic and clinical data, including age, gender, previous medical history, initial vital signs, severity, and laboratory values on admission, and interventions were retrieved from the registry. Sequential Organ Failure Assessment (SOFA) and Acute Physiology and Chronic Health Evaluation (APACHE) II scores were evaluated using the worst parameters within 24 h after ED arrival.

All laboratory data including lactate level were measured at the ED upon initial septic shock recognition. The normal range of lactate level was defined <2 mmol/L and we recorded the categorical variable of lactate normalization timing according to category (<6 h, <24 h, and did not normalize) until 24 h from shock recognition.

The primary clinical outcome of this study was the 28-day mortality rate. We evaluated the predictive ability of 6- and 24-h lactate normalization for 28-day mortality.

### Statistical analysis

Continuous variables were expressed as means ± standard deviation (SD) or as medians with interquartile range (IQR) if the assumption of a normal distribution was violated. Categorical variables were expressed as numbers and percentages. To analyze the baseline characteristics and laboratory examinations in the survivor and non-survivor groups, Student’s *t*-tests were used to compare the means of normally distributed continuous variables, whereas Mann–Whitney U-tests were used to compare non-normally distributed continuous data. Chi-squared or Fisher’s exact tests were used to compare categorical variables. Multivariate analyses were performed using logistic regression with a backward elimination method to evaluate the association between clinical factors including each 6- and 24-h lactate normalization and 28-day mortality. Variables with p-value < 0.05 in the univariate analysis were considered in the multivariate analyses. The results of the multivariate logistic regression analysis were reported as odds ratios (ORs) and 95% confidence intervals (CIs).

All tests in this study were two-sided, and *p*-values <0.01 were considered statistically significant. All statistical analyses were performed using IBM SPSS Statistics for Windows, version 20.0 (IBM Corp., Armonk, NY, USA).

## Results

**Fig 1:**
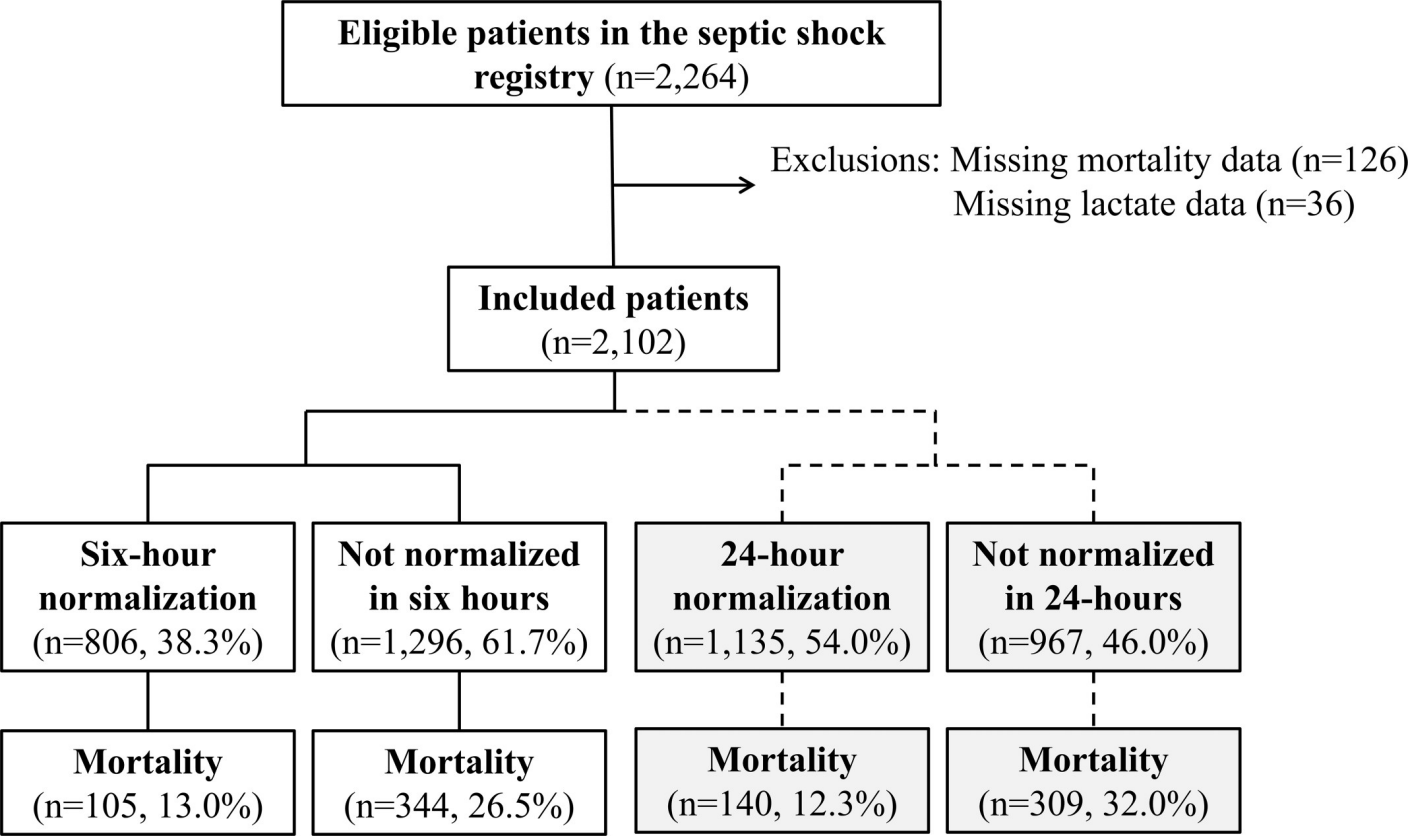
Diagram of the included patients.

During the study period, 2,264 eligible septic shock patients were enrolled in the registry. We excluded 162 patients with missing data, leaving 2,102 patients who were included in the present study. Of the included patients, 806 (38.3%) achieved lactate normalization within 6 h from recognition, with a mortality rate lower than that in the group that did not achieve normalization within 6 h (13.0% vs. 26.5%). In addition, 135 (1,135/2,102, 54.0%) patients achieved lactate normalization within 24 h from recognition; their mortality rate was also lower than that of the patients who did not achieve normalization within 24 h (12.3% vs. 32.0%) (Fig 1).

The overall mortality rate of included patients was 21.4%. Comparison of the survivor group (1,653 patients) to the non-survivor group (449 patients) revealed that the non-survivor group was significantly older (67.0 ± 13.5 vs. 70.2 ± 12.7 years, p<0.001), predominantly male (57.5% vs. 64.1%, p = 0.011), and had a higher prevalence of diabetes (29.5% vs. 34.3%, p = 0.048) and malignancy (21.8% vs. 28.5%, p = 0.003). The initial pulse rate was higher in survivor group (109.5 ± 24.9 vs. 106.8 ± 24.8 beats/min, p = 0.047). In the laboratory test results, creatinine, aspartate transaminase, initial lactate levels and C-reactive protein were higher in the non-survivor group (1.3 [0.9–2.0] vs. 1.6 [1.0–2.5], p<0.001; 38.0 [24.0–78.0] vs. 45.5 [27.3–104.8], p<0.001; 3.0 [1.8–4.9] vs. 4.7 [2.7–8.5], p<0.001, and 11.9 [4.2–21.2] vs. 14.6 [6.0–25.1], p = 0.001 respectively). However, hemoglobin was higher in survivor group (11.1 ± 2.5 vs. 10.6 ± 2.8, p<0.001). Finally, the SOFA score was also higher in non-survivor group (10.7 ± 4.1 vs. 7.7 ± 3.5, p-values <0.001) (Table 1).

**Table 1:** Baseline and clinical characteristics and laboratory findings.

28-day mortality	Total (n = 2,102)	Survivor (n = 1,653)	Non-survivor (n = 449)	*p*-value
Age, years	67.7 ± 13.4	67.0 ± 13.5	70.2 ± 12.7	<0.001
Male	1238 (58.9)	950 (57.5)	288 (64.1)	0.011
Past medical history				
Hypertension	865 (41.2)	677 (41.0)	188 (41.9)	0.727
Stroke	190 (11.5)	190 (11.5)	62 (13.8)	0.181
Diabetes	641 (30.5)	487 (29.5)	154 (34.3)	0.048
Cardiovascular disease	278 (13.2)	219 (13.2)	59 (13.1)	0.952
Chronic liver disease	250 (11.9)	191 (11.6)	59 (13.1)	0.357
Malignancy	489 (23.3)	361 (21.8)	128 (28.5)	0.003
Vital signs at shock recognition				
Systolic blood pressure, mmHg	99.5 ± 29.6	99.8 ± 30.0	98.5 ± 28.2	0.426
Diastolic blood pressure, mmHg	60.5 ± 19.3	60.6 ± 19.5	60.1 ± 18.2	0.642
Pulse rate, beats/min	108.9 ± 24.9	109.5 ± 24.9	106.8 ± 24.8	0.047
Laboratory findings				
White blood cell count, x 10^3^/μL	10.4 [5.2–17.1]	10.5 [5.5–17.0]	10.3 [4.2–17.1]	0.214
Hemoglobin, g/dL	11.0 ± 2.5	11.1 ± 2.5	10.6 ± 2.8	<0.001
Creatinine, mg/dL	1.4 [0.9–2.2]	1.3 [0.9–2.0]	1.6 [1.0–2.5]	<0.001
Aspartate transaminase, IU/L	40.0 [25.0–83.0]	38.0 [24.0–78.0]	45.5 [27.3–104.8]	<0.001
Alanine transaminase, IU/L	26.0 [15.0–54.0]	26.0 [15.0–53.0]	25.0 [14.0–59.0]	0.998
Initial lactate, mmol/L	3.3 [1.9–5.4]	3.0 [1.8–4.9]	4.7 [2.7–8.5]	<0.001
C-reactive protein, mg/dL	12.4 [4.6–21.9]	11.9 [4.2–21.2]	14.6 [6.0–25.1]	0.001
6 hour lactate normalization	806 (38.3)	701 (42.4)	105 (23.4)	<0.001
24 hour lactate normalization	1,135 (54.0)	995 (60.2)	140 (31.2)	<0.001
SOFA score	8.3 ± 3.8	7.7 ± 3.5	10.7 ± 4.1	<0.001

Values were expressed as means ± standard deviation, medians [interquartile range], or numbers (%). Abbreviation: SOFA, sequential organ failure assessment

Multivariate analysis of 6-h lactate normalization adjusted for age, sex, past medical history of diabetes and malignancy, pulse rate, hemoglobin, creatinine, aspartate transaminase, C-reactive protein and SOFA score which had p<0.05 in univariate analysis. However, we did not adjust initial lactate level because it may interact to the value of lactate normalization which was calculated with initial lactate level. As a result, the 6-h lactate normalization, age, malignancy history, initial pulse rate, hemoglobin, and SOFA score were independently associated with 28-d mortality (OR 0.58 [95% CI 0.45–0.75], p<0.001; OR 1.03 [95% CI 1.02–1.04], p<0.001; OR 1.53 [95% CI 1.17–2.00], p = 0.002; OR 1.01 [95% CI 1.01–1.02], p<0.001; OR 1.01 [95% CI 1.01–1.02], p<0.001, and OR 1.14 [95% CI 1.09–1.19], p<0.001, respectively). In addition, 24-h lactate normalization also showed independent association for the same factors (OR 0.42 [95% CI 0.33–0.54], p<0.001; OR 1.03 [95% CI 1.02–1.04], p<0.001; OR 1.49 [95% CI 1.13–1.95], p = 0.004; OR 1.01 [95% CI 1.01–1.02], p = 0.001; OR 0.91 [95% CI 0.87–0.96], p = 0.001, and OR 1.19 [95% CI 1.15–1.23], p<0.001, respectively) (Table 2). The p-values of Hosmer and Lemeshow tests in both logistic regression analyses were 0.221 and 0.871, respectively. And when we tested the collinearity with variance inflation factor, there was not any collinearity between independent variables in both analyses.

**Table 2:** Univariate and multivariate analysis of 6 and 24 hour lactate normalization.

Logistic Regression analysis	Univariate analysis OR [95% CI]	Multivariate analysis OR [95% CI]	p-value
Early lactate normalization			
6 hour lactate normalization	OR 0.42 [95% CI 0.33–0.53]	OR 0.58 [95% CI 0.45–0.75]	<0.001
Age	OR 1.02 [95% CI 1.01–1.03]	OR 1.03 [95% CI 1.02–1.04]	<0.001
Sex (Male)	OR 0.76 [95% CI 0.61–0.94]		
Diabetes	OR 1.25 [95% CI 1.00–1.56]		
Malignancy	OR 1.43 [95% CI 1.13–1.81]	OR 1.53 [95% CI 1.17–2.00]	0.002
Pulse rate, beats/min	OR 1.02 [95% CI 1.01–1.02]	OR 1.01 [95% CI 1.01–1.02]	<0.001
Hemoglobin, g/dL	OR 0.92 [95% CI 0.88–0.96]	OR 1.01 [95% CI 1.01–1.02]	<0.001
Creatinine, mg/dL	OR 1.13 [95% CI 1.06–1.20]		
Aspartate transaminase, IU/L	OR 1.00 [95% CI 1.00–1.01]		
C-reactive protein, mg/dL	OR 1.01 [95% CI 1.00–1.02]		
SOFA score	OR 1.23 [95% CI 1.20–1.27]	OR 1.14 [95% CI 1.09–1.19]	<0.001
Late lactate normalization			
24 hour lactate normalization	OR 0.30 [95% CI 0.24–0.37]	OR 0.42 [95% CI 0.33–0.54]	<0.001
Age	OR 1.02 [95% CI 1.01–1.03]	OR 1.03 [95% CI 1.02–1.04]	<0.001
Sex (Male)	OR 0.76 [95% CI 0.61–0.94]		
Diabetes	OR 1.25 [95% CI 1.00–1.56]		
Malignancy	OR 1.43 [95% CI 1.13–1.81]	OR 1.49 [95% CI 1.13–1.95]	0.004
Pulse rate, beats/min	OR 1.02 [95% CI 1.01–1.02]	OR 1.01 [95% CI 1.01–1.02]	<0.001
Hemoglobin, g/dL	OR 0.92 [95% CI 0.88–0.96]	OR 0.91 [95% CI 0.87–0.96]	0.001
Creatinine, mg/dL	OR 1.13 [95% CI 1.06–1.20]		
Aspartate transaminase, IU/L	OR 1.00 [95% CI 1.00–1.01]		
C-reactive protein, mg/dL	OR 1.01 [95% CI 1.00–1.02]		
SOFA score	OR 1.23 [95% CI 1.20–1.27]	OR 1.19 [95% CI 1.15–1.23]	<0.001

Multivariate analysis of each lactate normalization time were adjusted with Age, Sex, past history of diabetes, and malignancy, initial pulse rate, hemoglobin, creatinine, aspartate transaminase, C-reactive protein, and SOFA score, Abbreviation: OR, Odds Ratio; CI, Confidence interval; SOFA, sequential organ failure assessment

## Discussion

In this study, lactate normalization was an independent prognostic factor in septic shock patients. Achieving normalization within not only 6 h from shock recognition but also within 24 h (delayed achievement) were independently associated with decreased mortality. To our knowledge, this is the first report on delayed lactate normalization. This result may indicate that lactate normalization has roles as an early prognostic marker as well as a therapeutic target. Therefore, physicians managing septic shock patients should make efforts to achieve lactate normalization though prolonged resuscitation.

Normalization of lactate has been recommended in patients with elevated lactate levels targeting resuscitation since the 2013 SSC guidelines[7]; however, previous studies reported evidence of lactate clearance rather than normalization. Jansen et al. reported that a lactate level decrease of at least 20% per 2 h for the first 8 h was associated with a 9.6% absolute reduction in mortality[5] and a meta-analysis showed that lactate clearance is a useful biomarker.[4] In the cardiac arrest patients who treated with extracorporeal cardiopulmonary resuscitation, although lactate clearance did not predict mortality, it could predict a good neurologic outcomes.[17] Moreover, lower delta-lactate at 24h after admission was associated with adverse outcomes in critically ill patients.[18] In the present study, we found that lactate normalization within 6 h of initial resuscitation significantly decreased mortality. A previous study that included septic shock patients with abnormal initial lactate level (≥2 mmol/L) who received at least two lactate measurements in the first 6 h of resuscitation in the ED reported comparable results between lactate clearance and normalization.[19] In their study, both lactate normalization and lactate clearance over 50% were associated with significantly increased survival (adjusted OR 5.2 [95% CI 1.7–15.8] and adjusted OR 4.0 [1.6–10.0], respectively). However initial lactate level and 10% lactate clearance were not associated with survival rate (adjusted OR 1.2 [95% CI 0.5–2.5] and adjusted OR 1.6 [0.6–4.4]).[19]

There are few studies on lactate normalization as a prognostic factor in septic shock.[3,19–21] A previous study showed that the effect of lactate clearance and normalization decreased the rate of persistent organ dysfunction at 48 h in pediatric sepsis patients. Although lactate clearance was not associated with a reduced persistent organ failure rate (RR 0.70 [95% CI 0.35–1.41]), lactate normalization was significantly associated (RR 0.46 [95% CI 0.29–0.73]). Moreover, in subgroup analysis of the elevated initial lactate group only, lactate normalization was associated with 48-h organ failure (RR 0.47 [95% CI 0.29–0.78]).[20] The ANDROMEDA-SHOCK study, recent randomized clinical trial also reported that a resuscitation strategy targeting normalization of capillary refill time did not show effectiveness than targeting serum lactate level in 28-day mortality.[22] In our study, both 6- and 24-h lactate normalization was associated with a lower SOFA score (OR [22]0.88 [95% CI 0.86–0.91] and OR 0.86 [0.84–0.89]). Although we did not have long-term organ dysfunction data, the SOFA score, which was the maximum organ dysfunction score within 24 h from shock, was also associated with lactate normalization.

In sepsis patients, chronic liver disease and kidney failure cause hyperlactatemia due to impairment of lactate clearance.[1] A previous study reported liver dysfunction to be significantly associated with impaired lactate clearance and normalization during the early resuscitation of sepsis.[21] Our study also showed that chronic liver disease decreased 6- and 24-h lactate normalization rates (OR 0.45 [95% CI 0.33–0.61] and OR 0.60 [9 0.46–.78], respectively). However, chronic kidney disease was not associated with lactate normalization (OR 1.15 [95% CI 0.83–1.59]) and OR 1.04 [0.76–1.44]). Chronic liver and kidney disease did not differ between the survivor and non-survivor groups. Therefore, although chronic liver disease may make lactate normalization challenging, physicians should make efforts to achieve lactate normalization in survivors.

Zhang et al. reported that patients with lactate normalization had a significantly reduced hazard rate compared to those without normalization (log-rank test: p<0.05). They analyzed data from a public clinical database collected from the intensive care units (ICUs) of a university hospital. The population included all ICU patients with initial arterial blood lactate level >2 mmol/L. A Cox proportional hazard model was used. The median times for normalization in total, survivors, and non-survivors were 20.7 [9.6–43.2], 19.9 [9.3–40.7], and 24.7 [11.5–56.8] h, respectively, and the differences were statistically significant (p<0.001).[3] Although the populations differed from those in the present study, we also showed a significantly higher lactate normalization rate within 24 h for survivors than that in non-survivors (60.2% vs. 31.2%, p<0.001).

In our study, of 1,135 septic shock patients who achieved lactate normalization within 24 h from shock recognition, 806 achieved normalization within 6 h, while 329 did so within 6–24 h. The 28-day mortalities in these groups were 23.4% (105/806) and 10.2% (35/329), respectively, while that in the group of patients that did not achieve normalization within 24 h was 68.8% (309/967). During the early resuscitation period, various factors influence mortality in septic shock. However, after initial resuscitation lactate goes to more predictable factor. Our previous study comparing the timelines of lactate levels to predict mortality also showed the value of late lactate levels after 6 h (OR for 0, 2, 4, 6, and 12 h: 1.17 [95% CI 1.11–1.23], 1.23 [1.17–1.30], 1.30 [1.22–1.38], 1.33 [1.26–1.42], and 1.24 [1.19–1.30], respectively).[23] Although lactate normalization rates decreased from 38.3% (806/2,102) at 6 h to 25.4% (329/1,296) at 6–24 h, they were still high.

This study had several limitations. Since this study used data from a prospective multicenter registry, the data were fixed and additional data could not be collected. The data on lactate normalization were defined according to the first normalization time and were collected in three categories: within 6 h, within 24 h, and unable to achieve normalization. Thus, some patients developed re-hyperlactatemia after early lactate normalization. However, they also responded to early resuscitation. For the same reasons, lactate measurement time was not controlled. Some patients who achieved lactate normalization after 6 h from shock recognition might have already achieved normalization during initial resuscitation.

## Conclusions

Early lactate normalization within 6 h as well as delayed lactate normalization within 24 h from shock recognition was also independently associated with mortality. Lactate normalization may have a role in early risk stratification as well as a therapeutic target.

## Supporting information

S1 File Ryoo et al Lactate normalization PLOS ONE.sav. The minimal data set of this research.
(SAV)
